# Spontaneous helix formation in non-chiral bent-core liquid crystals with fast linear electro-optic effect

**DOI:** 10.1038/ncomms11369

**Published:** 2016-05-09

**Authors:** Sithara P. Sreenilayam, Yuri P. Panarin, Jagdish K. Vij, Vitaly P. Panov, Anne Lehmann, Marco Poppe, Marko Prehm, Carsten Tschierske

**Affiliations:** 1 Department of Electronic and Electrical Engineering, Trinity College Dublin, The University of Dublin, Dublin 2, Ireland; 2 School of Electrical and Electronic Engineering, Dublin Institute of Technology, Dublin 8, Ireland; 3 Department of Chemistry, Martin Luther University Halle-Wittenberg, D-06120 Halle, Germany

## Abstract

Liquid crystals (LCs) represent one of the foundations of modern communication and photonic technologies. Present display technologies are based mainly on nematic LCs, which suffer from limited response time for use in active colour sequential displays and limited image grey scale. Herein we report the first observation of a spontaneously formed helix in a polar tilted smectic LC phase (SmC phase) of achiral bent-core (BC) molecules with the axis of helix lying parallel to the layer normal and a pitch much shorter than the optical wavelength. This new phase shows fast (∼30 μs) grey-scale switching due to the deformation of the helix by the electric field. Even more importantly, defect-free alignment is easily achieved for the first time for a BC mesogen, thus providing potential use in large-scale devices with fast linear and thresholdless electro-optical response.

## Abstract (web summary)

Liquid crystals that consist of rod-like molecules currently dominate the materials used in industry for display and photonic applications. Here, the authors demonstrate electro-optic grey-scale switching based on the spontaneous formation of a short-pitch helix in a tilted smectic phase of achiral bent-core molecules.

## Graphical abstract

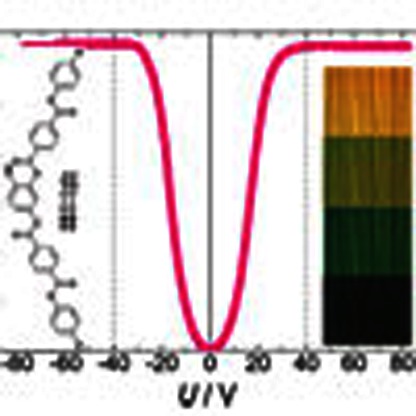

Liquid crystals (LCs) are of significant importance for display applications, photonics, sensors, communication technologies and data processing systems. LCs used in industry, so far, are predominately nematics that consist of simple rod-like molecules^1^. Disadvantages of nematic devices are their limited response time, failure to achieve RGB colour sequential display and a limited image grey scale. Faster switching is achieved with LC phases formed by chiral molecules, as, for example, blue phases^2^, ferroelectric and antiferroelectric switching synclinic tilted (SmC*, FLC) or anticlinic tilted smectic phases (SmC_A_*, AFLC)^3^. A new class of compounds with ferroelectric and antiferroelectric LCs is built from achiral bent-core (BC) molecules and has engendered great scientific interest in recent years^4^ due to a range of fascinating phenomena arising from the interplay of polarity and chirality^5^,^6^,^7^. Unlike rod-like LCs, the BC compounds, even being achiral, may exhibit spontaneous polarization in the orthogonal (SmA–like)^8^ and tilted (SmC–like) smectic phases^4^,^9^. In contrast to orthogonal BC phases^10^,^11^, electro-optical effects can possibly be used for applications in tilted smectic phases of BCLC have not so far been reported, although electro-optical switching has been observed^4^,^9^,^12^. A major reason is the impossibility to align these BC SmC phases. Besides these technological relevant aspects, spontaneous emergence of chirality in the tilted smectic phases of achiral BCLC is of prime general scientific importance^13^,^14^. Chirality in the LC phases of BCLC results from the combination of tilt and polar order in the smectic phases (Supplementary Fig. 1)^9^ and was found for the so-called dark conglomerate phases^5^,^13^, representing strongly distorted smectic phases with sponge-like structure^15^ or formed by helical nano-filaments^16^ and nano-size crystallites^17^. However, formation of smectic phases with helical superstructure having a helix axis parallel to the layer normal was not yet observed in the LC phases of any achiral BC mesogen^5^,^6^,^18^.

Here we report the first experimental confirmation of formation of a helix in a polar SmC phase of achiral BC molecules with a pitch much shorter than the optical wavelength, showing fast (∼30 μs) grey-scale switching due to the deformation of the helix by the electric field. In contrast to other BC smectics, defect-free alignment is easily achieved, thus providing potential use in devices with fast linear and thresholdless electro-optical response.

## Results

### LC phases of compounds **1/** ***n***

**Figure 1:**
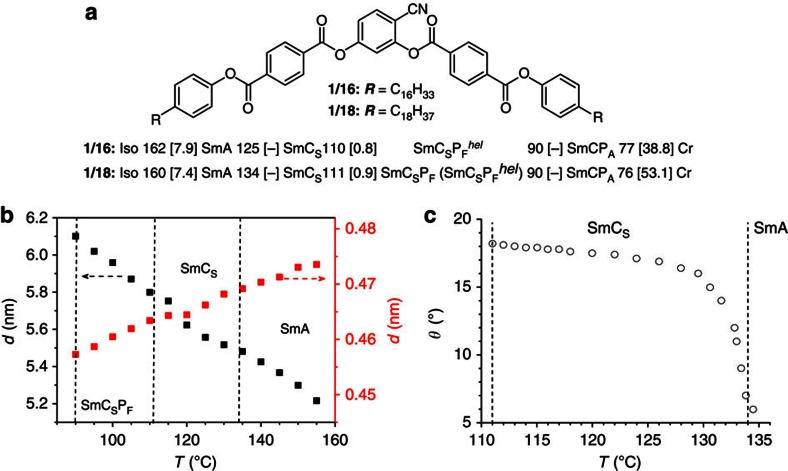
Molecular structure and LC phases of 1/*n*. (**a**) Molecular structure and the phase sequences with transition temperatures (*T* (°C)) and transition enthalpies (Δ*H* (kJ mol^−1^), in square brackets) as observed on cooling from the isotropic liquid state. Cr, solid crystal; Iso, isotropic liquid state; SmA, non-tilted and non-polar smectic (lamellar) LC phase; SmCP_A_, antiferroelectric switching polar SmC phase; SmC_S,_ paraelectric smectic phase with uniform (synclinic) tilt; SmC_S_P_F_ (synclinic ferroelectric LC phase, (Supplementary Fig. 1) and SmC_S_P_F_^*hel*^ helical synclinic ferroelectric phase with a short pitch and a helical axis lying parallel to the layer normal; SmCP_A_, antiferroelectric switching polar SmC phase. For **1/18**, the parentheses indicate that the SmC_S_P_F_^*hel*^ phase is only formed after application of an electric field, whereas SmC_S_P_F_ is the stable ground state structure in this temperature range prior to the field being applied. (**b**) Dependence of the *d*-values of the small angle scattering (black, left; layer thickness *d* follows the de Vries-like behaviour in tilted smectics) and the maximum of the wide angle scattering (red, right) on temperature in the XRD patterns of the smectic phases of compound **1/18**. (**c**) Temperature dependence of the optical tilt *θ* in the SmC_S_ phase of compound **1/18** as measured by rotating the LC sample in a 9 μm planar cell under the polarizing optical microscope from the dark to the bright state (under an applied square-wave voltage of 40 V at 10 Hz, low frequency ac voltage is applied to minimize the effects of ions during switching).

BC materials under study are the two compounds **1/*****n*** of the homologue series of 4-cyanoresorcinol bisbenzoates with terephthalate-based wings and long *n*-alkyl chains (***n***=16, 18) on both sides (Fig. 1a)^11^,^19^,^20^,^21^. The synthesis of these compounds was performed as described in the Methods (Fig. 5). An identification of the phases, their transition temperatures and the enthalpy values for the two investigated compounds **1/*****n*** are given in Fig. 1a.

Transition temperatures were obtained on cooling under quasi-equilibrium condition with a rate of ∼1 K min^−1^, whereas Δ*H* was taken from the DSC cooling curves (10 K min^−1^, Supplementary Figs 2 and 3). On cooling a non-tilted and non-polar smectic (lamellar) LC phase (SmA) is formed first, which on further cooling transforms into a smectic phase with uniform (synclinic) tilt (SmC_S_). This phase transition is observed optically by the occurrence of a birefringent Schlieren texture in the homeotropically aligned (optically uniaxial) SmA phase. The SmC phase is paraelectric and in the case of compound **1/16** changes into a SmC_S_P_F_^*hel*^ helical synclinic ferroelectric phase with a short pitch and a helical axis lying parallel to the layer normal at *T*=110 °C. For **1/18**, a polar switching (synclinic ferroelectric) SmC_s_P_F_ phase (Supplementary Fig. 1) without helical superstructure is formed at this phase transition (at *T*=111 °C); the SmC_S_P_F_^*hel*^ phase is only formed after the application of an electric field, whereas SmC_S_P_F_ is the stable ground state structure in this temperature range prior to the electric field having being applied. On further cooling, an antiferroelectric switching polar SmC phase (SmCP_A_) is formed for both compounds below *T*=90 °C. The polarization current curves recorded for the smectic phases of **1/18** under an applied triangular wave voltage are shown in the Supplementary Figs 6 and 10 and are discussed in detail in the Supplementary Note 1.

X-ray diffraction (XRD) patterns of aligned samples of the smectic phases of compounds **1/16** and **1/18** are shown in the Supplementary Figs 4 and 5, respectively. The diffraction patterns of both compounds are very similar and those of **1/18** are described here as an example of the case. There is a diffuse wide angle scattering with a maximum being shifted on cooling from *d*=0.47 nm in the SmA phase to *d*=0.46 nm in the SmC_s_P_F_ phase (Fig. 1b, red dots). The diffuse character of the wide angle scattering confirms the LC state of the phases under discussion and the decreasing *d*-value indicates a growing packing density with decreasing temperature. A sharp small angle reflection corresponding to *d*=5.2nm appears in the SmA phase. It corresponds to 0.7 molecular length (*L*_mol_=7.4 nm in the most extended conformation between the ends of the alkyl chains in *all-trans* conformation), in line with a monolayer smectic phase. The *d*-value of the layer reflection increases with decreasing temperature, due to the alkyl chain stretching with growing packing density (Fig. 1b, black dots and Supplementary Table 2). The *d*-value even continues its growth at the SmA–SmC transition and in the SmC_s_-range, reaching *d*=5.8–6.0 nm in the SmC_s_P_F_ phase. This means that, the effect of increasing packing density by alkyl chain stretching on the layer spacing is larger than the effect of developing the tilt. Indeed in the SmC_s_ phase an optical tilt of 18° is measured (Fig. 1c), which is relatively small compared with other bent-core mesogens where it is typically in the range of 35–45° (refs 5, 6, 18). Though the tilt is clearly confirmed by optical investigations, it is not evident from the XRD patterns, neither from the temperature-dependent development of the layer spacing nor from the positions of the wide angle with respect to the small angle scatterings in the two-dimensional (2D) XRD patterns of aligned samples, which is nearly orthogonal (90±5°) at all temperatures (Supplementary Fig. 5). This indicates that the optical tilt of ∼18° in the SmC_s_ phases mainly results from the tilt of the aromatic cores, whereas the more disordered aliphatic chains should be less tilted and preferably oriented opposite to the tilt direction of the aromatic cores. Similar results were obtained for compound **1/16** (Supplementary Fig. 4 and Supplementary Table 1), which have an even smaller optical tilt of 14°. This small tilt is assumed to be mainly due to the electron deficit terephthalate-based structure of the two rod-like wings attached to the 4-cyanoresorcinol core (Fig. 1a), which is known to support the formation of non-tilted or weakly tilted smectic phases^22^,^23^.

### Electro-optical investigations

**Figure 2:**
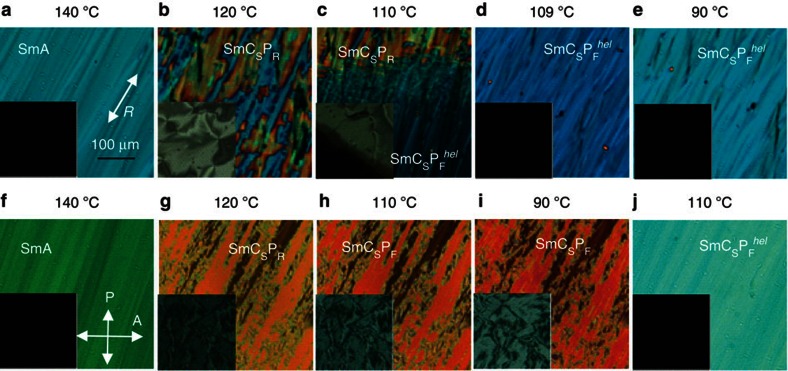
Field-induced textural changes. Polarizing microphotographs (**a**–**e**) of compound **1/16** and (**f**–**i**) compound **1/18** of 6.5 and 8 μm planar cells, respectively, taken on cooling from the isotropic phase. The insets show the corresponding textures of a 6.8 μm homeotropic cell under the same conditions; **a**–**i** were taken in the absence of an external field, and (**j**) of **1/18** is obtained on the removal of the field after the cell was subjected to a square wave field (40 V_pp_, *f*=110 Hz) at *T=*110 °C (**h**) with a corresponding homeotropic texture shown in the inset; ***R*** is the rubbing direction.

Figure 2 presents the polarizing microphotographs in planar cells for **1/16** in Fig. 2a–e and for **1/18** in Fig. 2f–i, these are taken on cooling from the isotropic phase in the absence of an electric field. The planar cells were rotated at an angle *α*=45°, where *α* is the angle between the rubbing direction, ***R*** and polarizer axis, ***P***. The textures of the homeotropic cells are shown in the insets of Fig. 2. At 140 °C (Fig. 2a,f) typical textures of both samples in planar cells correspond to a conventional uniaxial SmA phase with the optical axis lying along the rubbing direction ***R***. The homeotropic textures show perfect extinction, due to the orthogonal molecular organization in the layers (Fig. 2a,f, insets). On further cooling, the uniform texture breaks down into two sets of domains with an optical axis making an angle *±θ*_app_ with ***R*** (**1/16**: Fig. 2b; **1/18**: Fig. 2g), indicating the onset of a tilt which grows with a decrease in temperature (Fig. 1c). The homeotropic textures show typical Schlieren textures due to this tilt (insets in Fig. 2b,g), in line with the formation of a synclinic SmC_S_ phase. The SmC_S_ phases, occurring in the temperature range between 134 and 111 °C for compound **1/18** and between 125 and 110 °C for the shorter homologue **1/16**, do not respond optically to the electric field. However, under a triangular wave voltage a single polarization current peak develops on approaching the phase transition at *T*=110/111 °C, indicating the continuous growth of polar domains^24^, in line with dielectric results (Supplementary Fig. 7). For both compounds, the colour of the texture of the planar cell gradually changes due to an increase in the birefringence following a typical increase in the orientational order parameter on cooling. On further cooling the cell with the compound **1/16** shows a transition to a uniaxial texture at *T*=110 °C (Fig. 2c,d), which looks rather similar to SmA but with a lower birefringence of the planar sample than in SmA (compare Fig. 2a,d). This uniaxial phase, designated as helical SmC_S_P_F_^*hel*^ with a very short pitch persists down to *T*=90 °C. In the phase designation ‘P_F_' stands for a uniform polar direction (ferroelectric order) and superscript ‘*hel*' indicates the helical superstructure.

In contrast to the compound **1/16,** the textures of the planar cell for **1/18** do not show any principal changes at the phase transition at *T*=111 °C, (Fig. 2g,h) while the colour of the planar cell gradually changes (Fig. 2g–i) due to a continuous increase in the birefringence. However, at 111 °C weak electro-optical switching appears in response to the external electric field (*E*=0.9 V μm^−1^) similar to the observations reported by Eremin *et al*.^25^. On applying positive electric field, the optical axis of one type of domains switches to the right and the second domain switches to the left. The opposite is valid when negative electric field is applied. These features are typical for SmC_S_P_F_ phases (Supplementary Fig. 8j–m).

The application of a square-wave electric field of amplitude 3–4 V μm^−1^ to the SmC_s_P_F_ phase of **1/18** causes an irreversible structural transition, that is, on the removal of the electric field the texture does not return to the initial state (Fig. 2h) but forms a uniaxial, low-birefringence texture (Fig. 2j) similar to the SmC_S_P_F_^*hel*^ phase (Fig. 2d) observed in the compound **1/16** without application of an electric field (Supplementary Fig. 8a–i for details of the field-induced textures of **1/18**). Both textures are not only optically identical but also show similar electro-optical and other characteristics. Subsequent results and discussions are focused on **1/18** while at the same time these are applicable to **1/16** as well. It should be noted that textures of both samples on cooling in the absence of electric field show almost perfect planar alignment (Fig. 2a,f) unlike textures obtained in previous studies of BC mesogens^5^,^6^, where they consisted of focal conic defects. Such a good alignment had not previously been obtained.

**Figure 3:**
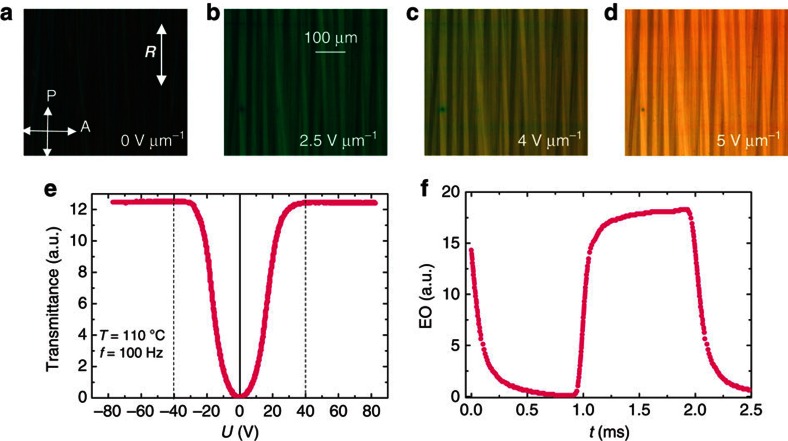
Electro-optics in the SmC_S_P_F_^*hel*^ phase of 1/18. Electro-optical investigations performed at 110 °C on a 8 μm planar cell. **a**–**d** show polarizing micrographs with sample in the field-induced SmC_S_P_F_^*hel*^ phase (at *T=*110 °C) taken at *α*=0° for different values of the electric field. *R* is the rubbing direction. **e** shows the optical response to an applied triangular voltage of ±80 V_pp_, (±10 V μm^−1^) at 100 Hz and **f** the electro-optical response to an applied square-wave 70 V_pp_ voltage, for this experiment *α*=22.5°.

Figure 3a–d shows the polarizing micrographs of **1/18** on a 8 μm planar cell in the field-induced helical SmC_S_P_F_^*hel*^ state, which exhibits the so-called V-shape switching. On the application of an electric field, the optical axis switches by a voltage-dependent angle *±θ* (V) from the rubbing direction with a corresponding gradual increase in the transmittance and birefringence (Fig. 3a–e), which saturates at 5 V μm^−1^ with a uniform texture (Fig. 3d). A large value of the birefringence Δ*n*_0_ as in the non-helical virgin cell (Fig. 2h) is obtained.

Optical investigation of the switching process shows hysteresis-free and fully continuous EO response (Fig. 3e,f), apparent switching angle and the effective birefringence as a function of the field (Supplementary Fig. 8). This kind of continuous and thresholdless switching in tilted smectics was previously observed in helix-free and high *P*_**S**_ ferroelectric SmC* phases^26^, de Vries materials^27^,^28^ and FLC materials with short helical pitch due to electric field-induced deformed helix (DHFLC)^29^, all requiring non-racemic chiral molecules, whereas the molecules considered here are achiral.

The remarkable feature of the SmC_S_P_F_^*hel*^ phase is a texture of the homeotropic cell (Fig. 2j, inset) which shows a perfect extinction independent of the cell rotation angle between the crossed polarizers. This texture is similar to the uniaxial SmA phase (Fig. 2f), while all polar or non-polar SmC subphases show Schlieren textures (Fig. 2g–i, insets). The optically uniaxial structure in tilted phases is only possible for de Vries phases, SmC_α_ or helical structures with a helical pitch shorter than the wavelength of light. Both, de Vries and SmC_α_ phases, are very unlikely to exist because they both exhibit a strongly temperature-dependent saturation voltage^27^, while in our study this is almost temperature independent over a relatively large temperature range of 20 K (Supplementary Fig. 9). Thus, we conclude that the texture in Fig. 3 corresponds to a short-pitch helical structure of the SmC_S_P_F_^*hel*^ phase.

Let us consider the physical reasons for the formation of a helix in non-chiral materials. According to the simple phenomenological theory developed by Pikin and Indenbom^30^, the helical pitch in chiral SmC* phase can be expressed as:


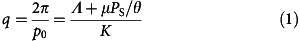


where *μ* is the flexoelectric coefficient, *P*_S_ is the spontaneous polarization, *θ* is molecular tilt angle, *K* is the effective elastic constant and *Λ* is Lifshitz invariant, which is responsible for the formation of the helix of a certain sense due to the molecular chirality. This means that chiral SmC* phase may form a helical structure even in the absence of spontaneous polarization. The existence of non-zero polarization shortens the helical pitch, while the sense of the helix is determined by the sign of *Λ*. In case of non-chiral molecules (*Λ=0*), the helical structure can be formed exclusively by soft matter with large spontaneous polarization. In other words, the formation of the helix reduces the electrostatic energy but increases the excess of elastic energy and the formation of helix of pitch *p*_0_*=2πKθ/μP*_S_ is a compromise between these two competing terms. In the absence of the Lifshitz invariant, the two helices of opposite sense are equi-probable and this is being observed in our sample. The formation of very short sub-optical pitch (Supplementary Fig. 8) in our case can be explained by large *P*_S_ value (∼300 nC cm^−2^), a moderate tilt angle (∼23°) (not shown in the Figure) and a reduced effective elastic constant due to the vicinity of anticlinic phase at a lower temperature.

It is known that the synchronization of helical conformers of non-chiral (but transiently chiral) molecules leads to spontaneous mirror symmetry breaking^31^, providing the chirality which determines the sign of *Λ.* Therefore, once formed, the helical structure stabilizes one of two possible molecular conformations due to the diastereomeric coupling between the helix sense and molecular conformations, that is, *Λ*≠0 and this additionally strengthens the helical organization with 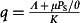 and together with next-nearest-neighbor interactions^32^ further shortens the helix pitch, formed originally due to *P*_**S**_. The existence of in-layer spontaneous polarization is also important in other liquid crystalline systems, for example, formation of ferrielectric subphases in AFLCs^33^.

The observed electro-optical response to the applied electric field is similar to that of the deformed helical structure in SmC* phases of highly chiral LCs (DHF effect)^29^, which had attracted enormous interest at the time of its discovery for a potential application to fast-response LC devices with a tunable grey-scale. For compounds **1/*****n*** in the SmC_S_P_F_^*hel*^ phase, the switching time is found to be ∼30–40 μs and the contrast ratio of the electro-optical switching was found to lie between 200 and 300. It should be noted that all previously reported DHFLC studies were conducted only on chiral SmC* phases of permanently chiral molecules with a large enough strength of the chirality. In contrast, the compounds used here are achiral and consequently these are more easily accessible when compared with highly chiral FLCs.

Due to the larger tilt, the layer coupling is stronger for **1/18**, which stabilizes the non-helical synclinic SmC_S_P_F_ state where the helix is suppressed. In this case, a sufficiently strong electric field is required for its transition to the helical SmC_S_P_F_^*hel*^ structure. The electric field improves ordering in the layers, leading to denser molecular packing, which is thought to support chirality synchronization of chiral conformers, thus supporting helix formation^31^. The SmC_S_P_F_^*hel*^ state once formed appears to be stable in both homeotropic and planar cells. For both compounds **1/16** and **1/18,** the antipolar coupling increases with decreasing temperature, reaching a critical value at ∼90 °C, when the SmCP_A_ phase is formed.

### Atomic force microscopy

**Figure 4:**
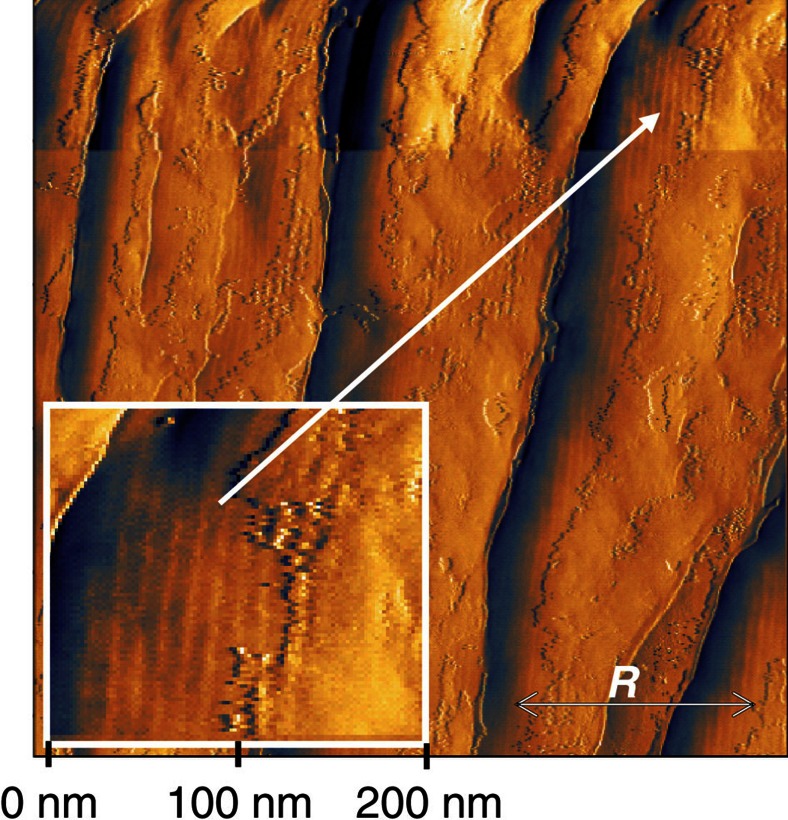
AFM image of the SmC_S_P_F_^*hel*^ phase in a planar cell. The 8 μm planar cell filled with **1/18** is used for AFM study. The helical (SmC_S_P_F_^*hel*^) phase at 105 °C was supercooled to the glassy state by immersing it into liquid nitrogen. Once supercooled, this material stays in the glassy state even at room temperature preserving the original structure of the phase under study. Then the glass plates of the LC cell were separated to enable the glassy material to be scanned by the AFM probe. The vertical stripes perpendicular to the rubbing direction ***R*** and smectic layer normal reveal a modulated structure corresponding to the helix.

An evidence for the modulated helical superstructure in the SmC_S_P_F_ phase is provided by the scanning of the sample by atomic force microscopy (AFM). The helical (SmC_S_P_F_^*hel*^) phase of **1/18** at 105 °C was super cooled to the glassy state by immersing it into liquid nitrogen. Once super cooled, this material stays in its glassy state^34^ even at room temperature thus preserving its original structure. Then the glass plates of the LC cell were separated to access the glassy material by the AFM probe. Figure 4 shows an AFM image of the glassy **1/18** sample in the SmC_S_P_F_^*hel*^ phase at room temperature. The vertical stripes perpendicular to the rubbing direction ***R*** and smectic layer normal reveal a modulated structure with a distance of ∼14 nm, corresponding to approximately three layers. This provides a direct evidence for the existence of short-pitch helical structure with an angle ∼150° between the **C**-directors in neighbouring smectic layers similar to the model suggested by Pikin *et al*.^35^. This pitch is at least 1 order of magnitude smaller than the minimal pitch detectable by optical techniques and this explains why the textures appear uniaxial in both homeotropic and planar geometries. This direct evidence supports our assumption of a helical structure in the SmC_S_P_F_^*hel*^ phase.

## Discussion

The structure of the SmC_S_P_F_ phase in relation to possible SmC_A_P_A_, SmC_α_ and de Vries structures is being considered here. It would seem that the SmC_A_P_A_–SmC_S_P_F_ transition in polar smectic phases of achiral BC mesogens is similar (structurally and electrically) to the electric field-induced SmC_A_*-to-SmC* transition in chiral ferroelectric LCs of rod-like molecules. This process occurs through appearances of ferroelectric domains as dark/bright fringes parallel to the smectic layer normal. Such fringes grow in size with an increase in the electric field applied along the smectic layer normal to complete a uniform field-induced ferroelectric state^36^, although there is a small linear pre-transitional effect^37^. Therefore, the intermediate grey-scale during SmC_A_–SmC transition is realized as a mixture of the antiferroelectric and ferroelectric domains (fringes) and electro-optic response must show both the voltage threshold and a strong hysteresis in the transmittance versus field. Most of the work on the BC systems as far as we are aware does not contain sufficient data on the intermediate states during the optical switching, with the exception of those given in refs 38, 39, where the threshold, hysteresis and the fringes have been observed. In contrast to these observations our data show hysteresis-free (Fig. 3e) and complete analogue response, fringe-less change in the switching and hysteresis-free apparent optical tilt angle and the effective birefringence with field. Blinov *et al*.^39^ have reported anomalously large pre-transitional effect in BC materials compared with the field-induced SmC_A_*–SmC* switching, which gives about 20–25% of total apparent optical angle. Nevertheless, the rest of switching goes through fringes-domains and so on. Therefore, the continuous switching observed in our study is unlikely to be due to the SmC_A_P_A_–SmC_S_P_F_ transition as was reported previously.

In the anticlinic SmC_A_P phase, value of the effective birefringence is Δ*n*_eff_(*θ*)=Δ*n*_0_ cos^2^ (2*θ*), where Δ*n*_0_ is the birefringence of the field-induced synclinic SmC_S_P state and for our case (*θ=*18°, *Δn*_0=_0.1095), Δ*n*_eff_ (*θ*) equals 0.088. The effective birefringence of short-pitch helix is the same as for the de Vries type of SmA/SmC* phases and depends on the tilt angle as: 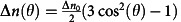 (ref. 40) and this gives a value of 0.094. The experimental value of 0.091 is exactly in between the two calculated values and hence both structures are plausible. Another strong argument in favour of de Vries and short-pitch helical structures is a texture of the homeotropic cell (Fig. 2j, inset) which shows a perfect extinction independent of the cell rotation angle between the crossed polarizers. This texture is similar to the uniaxial SmA phase (Fig. 2a), while all SmCP_A_ subphases show Schlieren (Fig. 2b–d, insets) textures. The optically uniaxial structure in tilted phases is only possible for de Vries phase or helical structures with a short sub-wavelength helical pitch. The first of the two, the de Vries phase is also ruled out because it shows a strongly temperature-dependent saturation voltage^27^, while in our study this is almost temperature independent over a relatively large range of 20 °C in temperature (Supplementary Fig. 9). In addition, the two polarization peaks were also recorded in the SmC*_α_ phases of chiral rod-like molecules^41^, having a similar short helical pitch superstructure as reported for the SmCP_α_ phase reported here for achiral BC molecules.

In conclusion, the spontaneous formation of a short-pitch helix in a tilted smectic phase of achiral BC LC is observed for the first time. This new helical LC phase (SmC_s_P_F_^*hel*^) exhibits a linear electro-optic effect and shows fast grey-scale switching due to the deformation of the helix. The fast switching with temperature-independent tilt angle makes these non-chiral BC LCs not only perspective candidates for high-speed, grey-scale application in LCDs, light modulators and electro-optical shutters, but also it provides a new mode of spontaneous mirror symmetry breaking. Helix formation emerges at the onset of long range polar order in weakly tilted smectic phases. It is proposed that developing layer chirality, being the result of the orthogonal combination of tilt, polar direction and layer normal^9^, couples with conformational chirality of the achiral, but transiently chiral BC molecules^13^,^31^. Interlayer helix formation obviously requires weak layer coupling as provided by compounds **1/16** and **1/18** having a relatively small synclinic tilt, whereas there is no indication of helix formation in any of the previously known polar SmC phases (B2 phases) of BC molecules, having much larger tilt angles and thus a stronger tilt correlation between the layers. Also in line with the proposed relation between layer coupling and helix formation, the SmC_s_P_F_^*hel*^ phase is a stable phase for **1/16**, whereas **1/18**, having a slightly larger tilt and consequently a stronger layer coupling compared with **1/16**, forms the SmC_s_P_F_^*hel*^ state only after application of a sufficiently strong electric field. Obviously, helix formation is associated with the nearly simultaneous emergence of tilt and polar order at the transition from the paraelectric SmC_s_ to the SmC_s_P_F_ phase. Possibilities of this phase behaving similarly to de Vries tilted smectic or tilted SmC_α_ phase have been considered but these possibilities are being ruled out.

## Methods

### Synthesis

**Figure 5:**
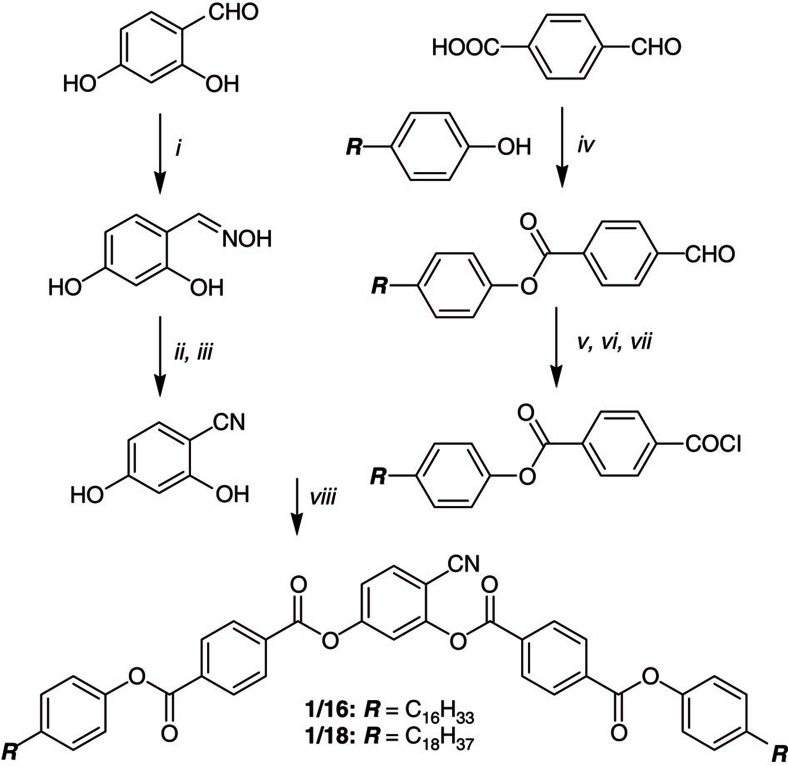
Synthesis of compounds 1/*n*. Reagents and conditions: (i) NH_2_OH·HCl, EtOH, Na_2_CO_3_, 20 °C, 6 h; (ii) Ac_2_O, reflux, 3 h; (iii) KOH, H_2_O, 20 °C, 72 h; (iv) DCC, DMAP, CH_2_Cl_2_, 20 °C, 24 h; (v) NaClO_2_, NaH_2_PO_2_, resorcinol; t-BuOH, 20 °C, 12 h; (vi) HCl, H_2_O, 20 °C, 20 min; (vii) SOCl_2_, DMF, reflux, 1 h; (viii) Et_3_N, cat. pyridine, CH_2_Cl_2_, reflux, 6 h.

Compounds **1/*****n*** were synthesized in-house according to the synthetic procedure shown in Fig. 5. Column chromatography was performed with silica gel 60 (63–200 μm, Fluka). Determination of structures and purity of intermediates and products was obtained by NMR spectroscopy (VARIAN Gemini 2000 and Unity Inova 500, VARIAN, all spectra were recorded at 27 °C). Microanalyses were performed using a CARLO Erba-CHNO 1102 elemental analyzer and MALDI-TOF MS measurements were performed on a Bruker Autoflex III system (Bruker Daltonics) operating in reflection and linear modes; the matrix solution was prepared by dissolving *trans*-2-[3-(4-*tert*-butylphenyl)-2-methyl-2-propenylidene]malononitrile (DCTB, purchased from Sigma-Aldrich) in THF with a concentration of 20 mg ml^−1^. The purity of all products was checked with thin layer chromatography (silica gel 60 F_254_, Merck). CHCl_3_/EtOAc mixtures and CHCl_3_/MeOH mixtures were used as eluents and the spots were detected by UV radiation.

For the synthesis of compounds **1/*****n*** the appropriate 4-(4-*n*-alkylphenoxycarbonyl)benzoic acid (synthesized in analogy to the procedures given in ref. 42) (0.8 mmol) was refluxed in excess thionylchloride (25 ml) under argon atmosphere, after 1 h the excess thionylchloride was removed under vacuum, then 4-cyanoresorcinol^43^ (54 mg, 0.4 mmol), triethylamine (0.07 ml, 0.52 mmol) and pyridine (0.05 ml) were dissolved in anhydrous CH_2_Cl_2_ (50 ml) and added to the acid chloride and stirring is continued under reflux for 6 h. The reaction mixture is then poured into aqueous 1 N HC1 (10 ml). The organic layer was separated and washed twice with saturated aqueous NaHC0_3_ solution. The combined aqueous layers were extracted with CH_2_C1_2_ (30 ml). The organic extracts were dried over anhydrous Na_2_SO_4_, filtered, and concentrated under vacuum. The crude products were purified by column chromatography using CHCl_3_/*n*-hexane (9/1 V/V) as eluent followed by two crystallizations from ethanol/chloroform (9/1 V/V) mixture to give the desired final compound (**1/*****n***) as colourless crystals.

### 4-Cyano-1,3-phenylene bis[4-(4-hexaadecylphenoxycarbonyl)benzoate] (**1/16**)

Yield 83%. ^1^H-NMR (500 MHz, CDCl_3_): *δ*/p.p.m. 8.40–8.30 (m, 8H, Ar-H), 7.84 (d, ^3^*J*_H–H=_8.5 Hz, 1H, Ar-H), 7.61 (d, ^4^*J*_H–H=_2.1 Hz, 1H, Ar-H), 7.38 (dd, ^3^*J*_H–H=_8.5 Hz, ^4^*J*_H–H=_2.1 Hz, 1H, Ar-H), 7.27–7.21 (m, 4H, Ar-H), 7.15 (m, 2H, Ar-H), 7.14 (m, 2H, Ar-H), 2.67–2.60 (m, 4H, Ar-CH_2_), 1.67–1.60 (m, 4H, CH_2_), 1.37–1.22 (m, 52H, CH_2_), 0.88 (t, ^3^*J*_H–H=_6.9Hz, 6H, CH_3_). ^13^C-NMR (101 MHz, CDCl_3_): *δ*/p.p.m. 164.08, 163.04, 162.72, 154.52, 153.22, 148.56, 148.53, 140.95, 140.89, 134.91, 134.79, 134.06, 132.55, 132.16, 130.60, 130.45, 130.38, 129.39, 129.37, 121.09, 121.06, 120.09, 117.23, 114.51, 104.57, 35.53, 32.06, 31.58, 29.83, 29.81, 29.73, 29.64, 29.49, 29.43, 22.83, 14.25. MALDO-TOF MS (*m/z*) [M+Na]+ calcd. for C_67_H_85_NO_8_Na, 1054.6; found 1054.6; analysis (calcd. for C_67_H_85_NO_8_) C 77.72 (77.95), H 8.13 (8.30), N 1.38 (1.36).

### 4-Cyano-1,3-phenylene bis[4-(4-hexaadecylphenoxycarbonyl)benzoate] (**1/18**)

Yield 58%. ^1^H-NMR (500 MHz, CDCl_3_): *δ*/p.p.m. 8.40–8.25 (m, 8H, Ar-H), 7.82 (d, ^3^*J*_H–H=_8.6 Hz, 1H, Ar-H), 7.59 (d, ^4^*J*_H–H=_2.2 Hz, 1H, Ar-H), 7.36 (dd, ^3^*J*_H–H=_8.5 Hz, ^4^*J*_H–H=_2.2 Hz, 1H, Ar-H), 7.23 (m, 4H, Ar-H), 7.13 (m, 2H, Ar-H), 7.12 (m, 2H, Ar-H), 2.67–2.54 (m, 4H, Ar-CH_2_), 1.71–1.54 (m, 4H, CH_2_), 1.38–1.18 (m, 60H, CH_2_), 0.86 (t, ^3^*J*_H–H=_7.0 Hz, 6H, CH_3_). ^13^C-NMR (126 MHz, CDCl_3_): *δ*/p.p.m. 164.20, 163.13, 162.81, 154.56, 153.24, 148.57, 148.54, 141.01, 140.95, 134.89, 134.77, 134.12, 132.54, 132.15, 130.63, 130.48, 130.42, 130.42, 129.44, 129.42, 121.11, 121.08, 120.14, 117.26, 114.54, 104.51, 77.24, 35.39, 31.91, 31.46, 29.69, 29.67, 29.66, 29.59, 29.50, 29.35, 29.28, 22.68, 14.10. MALDO-TOF MS (*m/z*) [M+Na]+ calcd. for C_71_H_93_NO_8_Na, 1110.7; found 1110.7; analysis (calcd. for C_71_H_93_NO_8_) C 78.20 (78.34), H 8.57 (8.61), N 1.28 (1.29).

### Optical and calorimetric investigations

Phase transitions were determined by polarizing microscopy (Leica DMR XP) in conjunction with a heating stage (FP 82 HT, Mettler) and controller (FP 90, Mettler) and by differential scanning calorimetry (DSC-7, Perkin Elmer) at heating/cooling rates of 10 K min^−1^ (peak temperatures).

### X-ray diffraction

XRD patterns of the aligned samples were recorded with a 2D detector (HI-STAR, Siemens or Vantec 500, Bruker). Ni-filtered and pin hole-collimated Cu-K_α_ radiation was used. Alignment was achieved by slow cooling (0.1 K min^−1^) of a small droplet of the compound on a glass plate and took place at the sample–air interface. The sample to detector distance was 9.0 cm and 26.8 cm for the wide angle and small angle measurements, respectively, and the exposure time was 15 min.

### Sample preparation for optical and electro-optical studies

The substrates of the planar cells for investigating the electro-optic response are coated with polymer RN 1175 (Nissan chemicals, Japan) and the coated surfaces are subsequently rubbed with a rotating commercial rubbing machine. The indium tin oxide (ITO)-coated glass substrates for the homeotropic cells are further coated with AL60702 polymer (JSR Korea). The gap between the electrodes for the in-plane field is 80 μm. The cell thickness was controlled by Mylar spacers of different thicknesses and the cell separation was measured by the optical interference technique. LC cells of these samples are studied using polarizing optical microscope (Olympus BX 52) equipped with an INSTEC's hot stage, temperature is controlled by Eurotherm 2,604 controller. The system is designed to obtain a temperature stabilization of the sample to within ±0.02°.

### Dielectric spectroscopy

Dielectric relaxation measurements over a frequency range 1 Hz–10 MHz were performed using a broadband Alpha High Resolution Dielectric Analyser (Novocontrol GmbH, Germany). The glass substrates coated with low sheet resistance (20 Ω per square) ITO electrodes were used to make cells. The sheet resistance of the cell substrates is relatively low and thus the peak frequency due to the sheet resistance of the ITO in series with the capacitance of the cell is shifted to a frequency much higher than 1 MHz. The experimental set-up is calibrated by the prior measurements of the capacitance of the empty cell. The measurement is carried out under the application of weak field 0.1 V μm^−1^. The temperature of the sample is stabilized within ±0.05 °C. The dielectric spectra are analysed using Novocontrol WINDETA programme. Temperature dependence of the dielectric strength (*δɛ*) and the relaxation frequency (*f*_R_), are obtained by fitting the dielectric spectra to the Havriliak–Negami (H–N) equation:


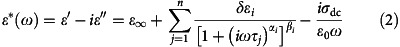


Here *ɛ*_∞_ is the high frequency dielectric permittivity depending on the electronic and atomic polarizability, *j* is the number of relaxation processes required for the fit and it varies from 1 to *n*, *ω*=2*πf* (frequency in Hertz) is the angular frequency, *ɛ*_0_ is the permittivity in free space, *τ*_*j*_ is the relaxation time of the *j*^th^ relaxation process, *δɛ*_*j*_ is the dielectric relaxation strength and *α*_*j*_ and *β*_*j*_, respectively, are the symmetric and asymmetric broadening parameters of the *j*^th^ process related to the distribution of relaxation time. The *σ*_dc_/*ɛ*_0_*ω* represents the contribution of dc conductivity to *ɛ*′′.

### Atomic force microscopy

The structure of helical phase was studied by AFM on a Veeco Nanoscope-IIIa system (Digital Instruments). The samples for AFM study were prepared by filling **1/18** LC material in planar cells. The helical (SmC_S_P_F_^*hel*^) phase at 105 °C was super cooled to the glassy state by immersing it into liquid nitrogen. Once super cooled, this material stays in the glassy state even at room temperature preserving the original structure. Then the glass plates of the LC cell were separated to access the glassy material by the AFM probe.

## Additional information

**How to cite this article:** Sreenilayam, S. P. *et al*. Spontaneous helix formation in non-chiral bent-core liquid crystals with fast linear electro-optic effect. *Nat. Commun.* 7:11369 doi: 10.1038/ncomms11369 (2016).

## Supplementary Material

Supplementary Information Supplementary Figures 1-10, Supplementary Tables 1-2, Supplementary Notes 1 and Supplementary References.
