# Supplementary material for: Spontaneous helix formation in non-chiral bent-core liquid crystals with fast linear electro-optic effect
Source: Nat Commun. 2016 May 9;7:11369. doi: 10.1038/ncomms11369 (PMC4865739; doi:10.1038/ncomms11369)
Supplement: Supplementary Information — Supplementary Figures 1-10, Supplementary Tables 1-2, Supplementary Notes 1 and Supplementary References. [file ncomms11369-s1.pdf]

Supplementary Information

Supplementary Figures

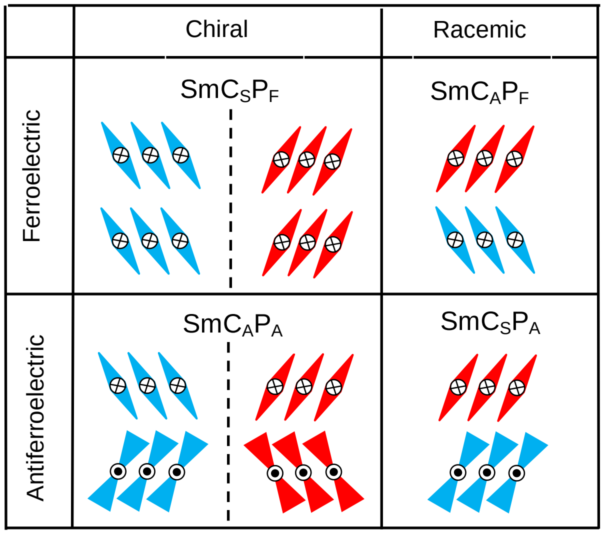

Supplementary Figure 1 │ Structures of the SmCP phases of bent-core mesogens. The four subtypes of tilted polar smectic phases resulting from the correlation of tilt direction and the polar direction (indicated by spots and crosses) of bent-core molecules in adjacent layers. The orthogonal combination of tilt and polar order leads to reduced C_{2v} symmetry and super structural chirality of the layers (color indicates chirality sense, dots and crosses indicate the polar direction pointing out of the projection plane and into the projection plane, respectively).

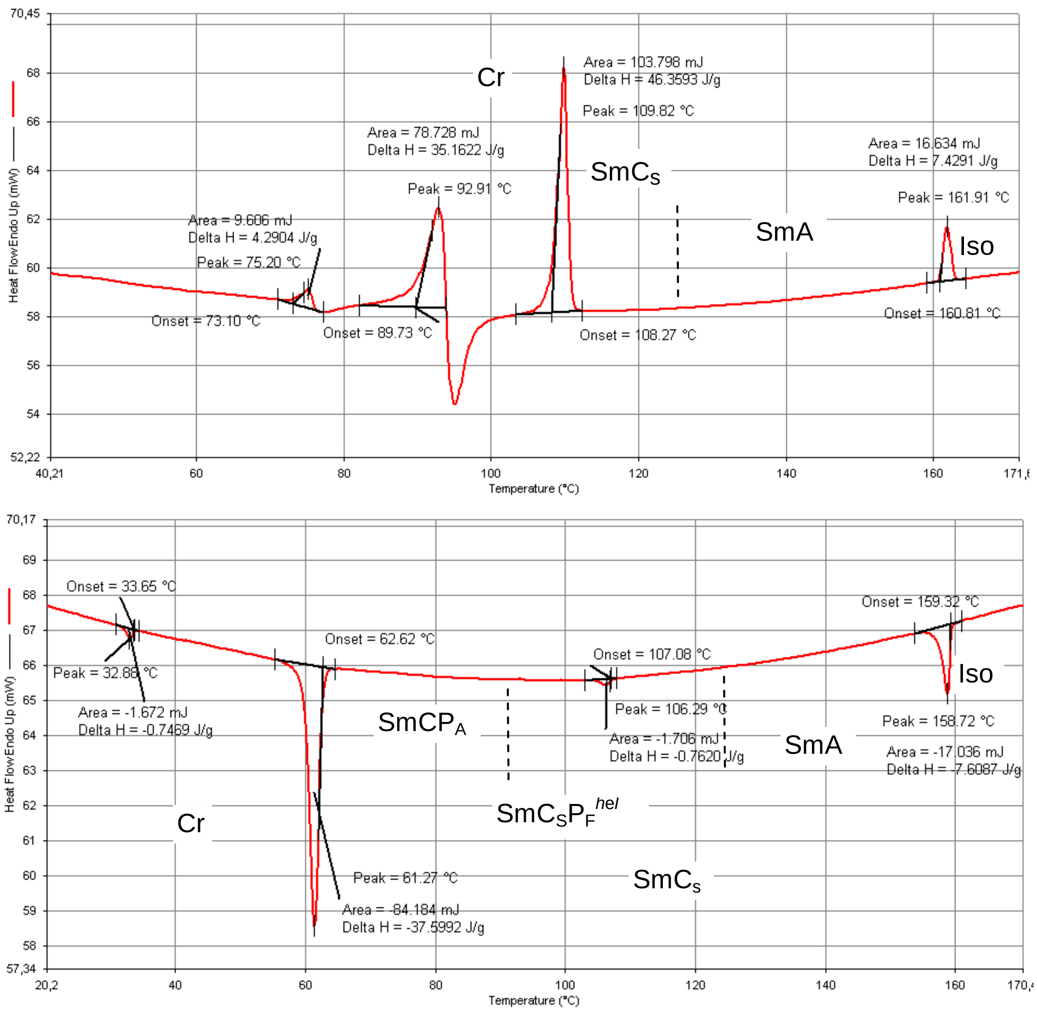

Supplementary Figure 2 │ Differential Scanning Calorimetry. DSC heating (top) and cooling (bottom) curves of **1/16** (10 K min^{-1}).

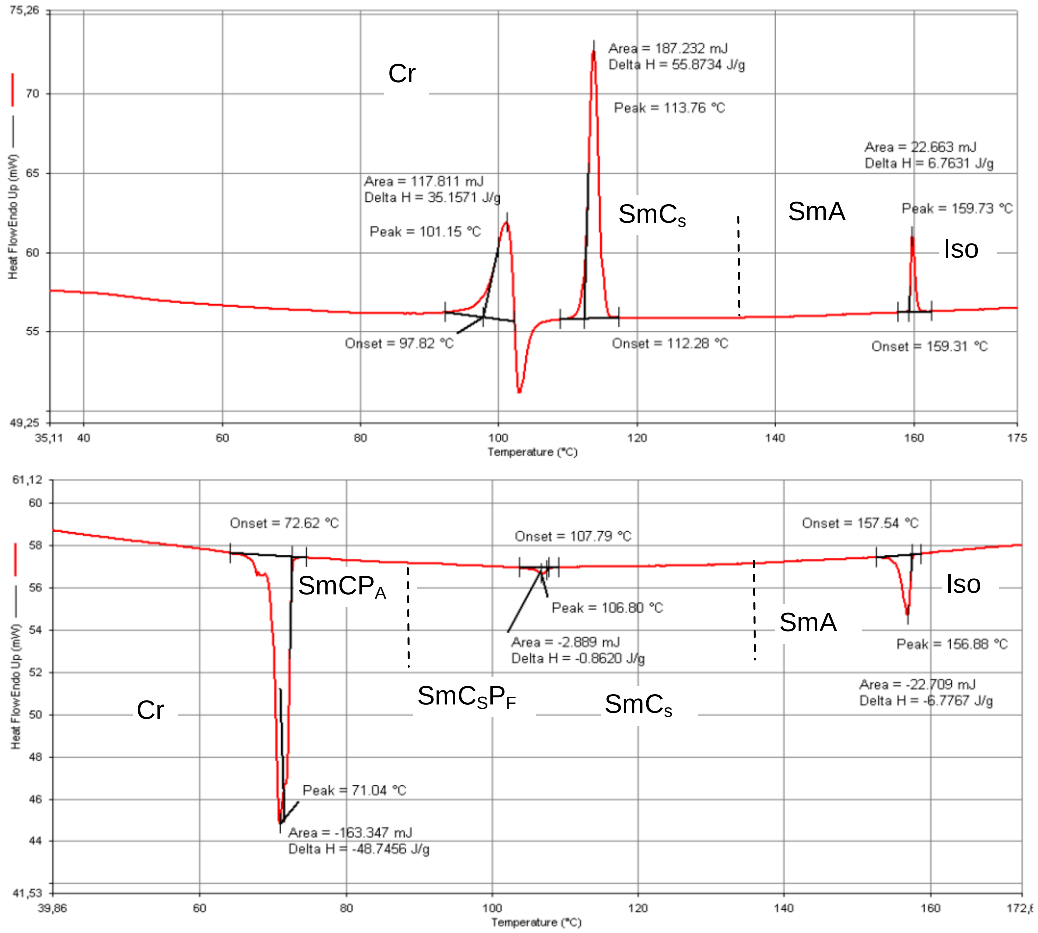

Supplementary Figure 3 │ Differential Scanning Calorimetry. DSC heating (top) and cooling (bottom) curves of **1/18** (10 K min^{-1}).

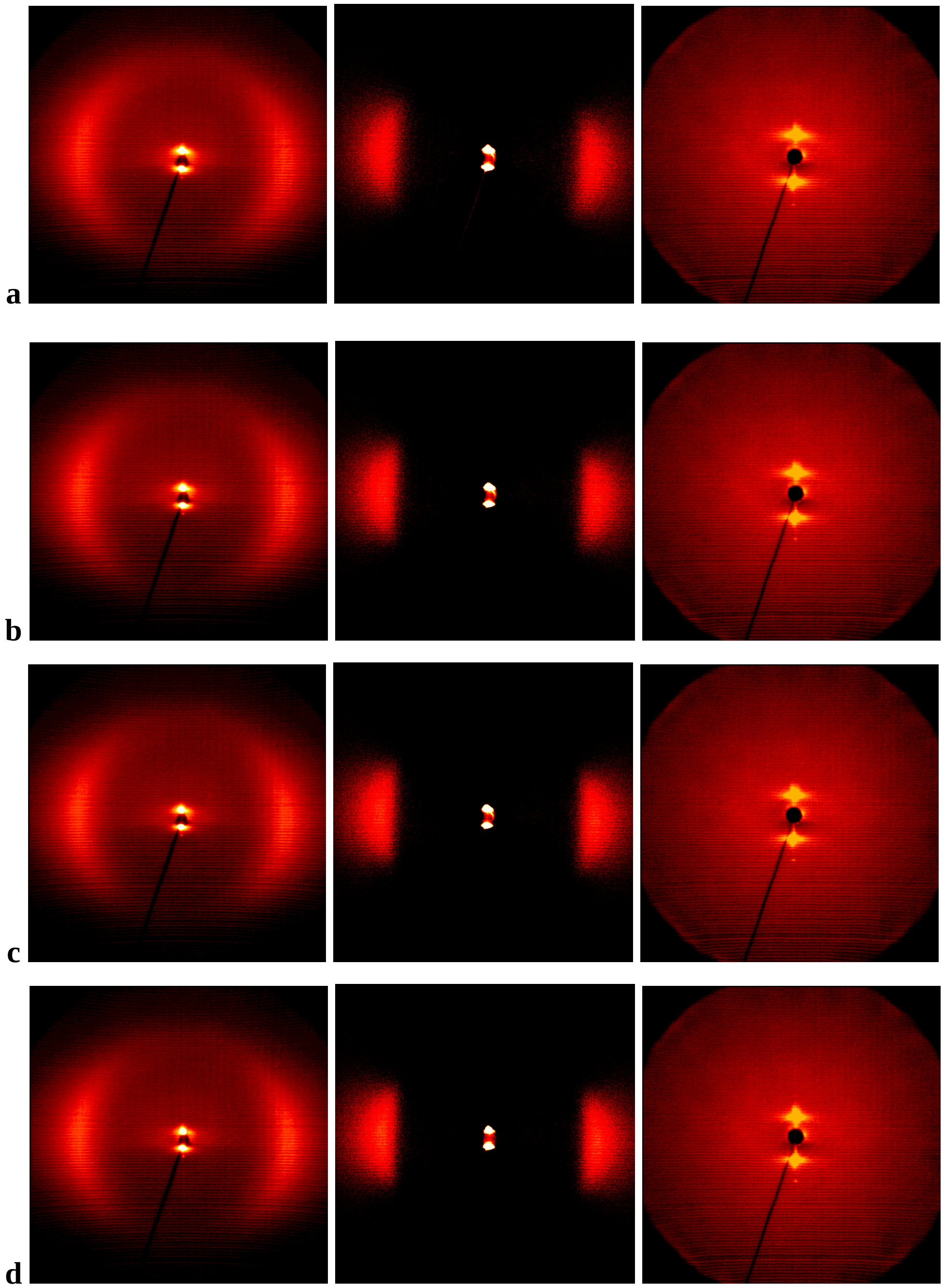

Supplemetary Figure 4 │ XRD patterns of an oriented sample of compound 1/16; (**a**) SmA at T = 140 °C; (**b**) SmC_S at T = 115 °C and (**c**) $SmC_SP_F^{hel}$ at T = 105 °C and (**d**) at T = 95 °C; the left row shows the original wide angle pattern, the middle row shows the wide angle scattering after subtraction of the scattering in the isotropic liquid state at T = 170 °C, the right row shows the small angle patterns.

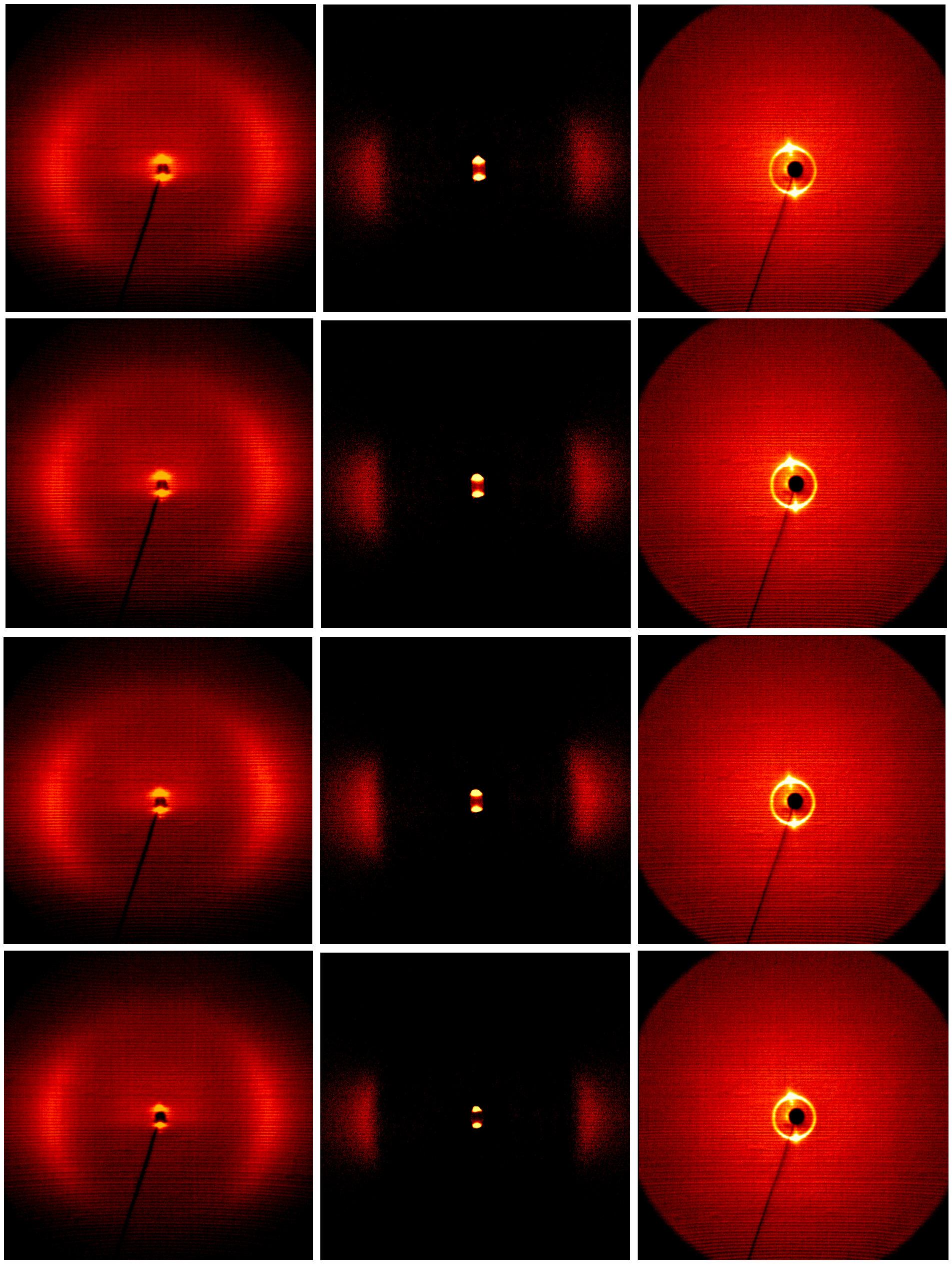

Supplemetary Figure 5 │ XRD patterns of an oriented sample of compound 1/18: (**a**) SmA at T = 140 °C, (**b**) SmC_S at T = 120 °C, (**c**) SmC_SP_F at T = 100 °C and (**d**) $SmCP_A$ at T = 80 °C; the left row shows the original wide angle pattern, the middle row shows the wide angle scattering after subtraction of the scattering in the isotropic liquid state at T = 170 °C, the right row shows the small angle patterns.

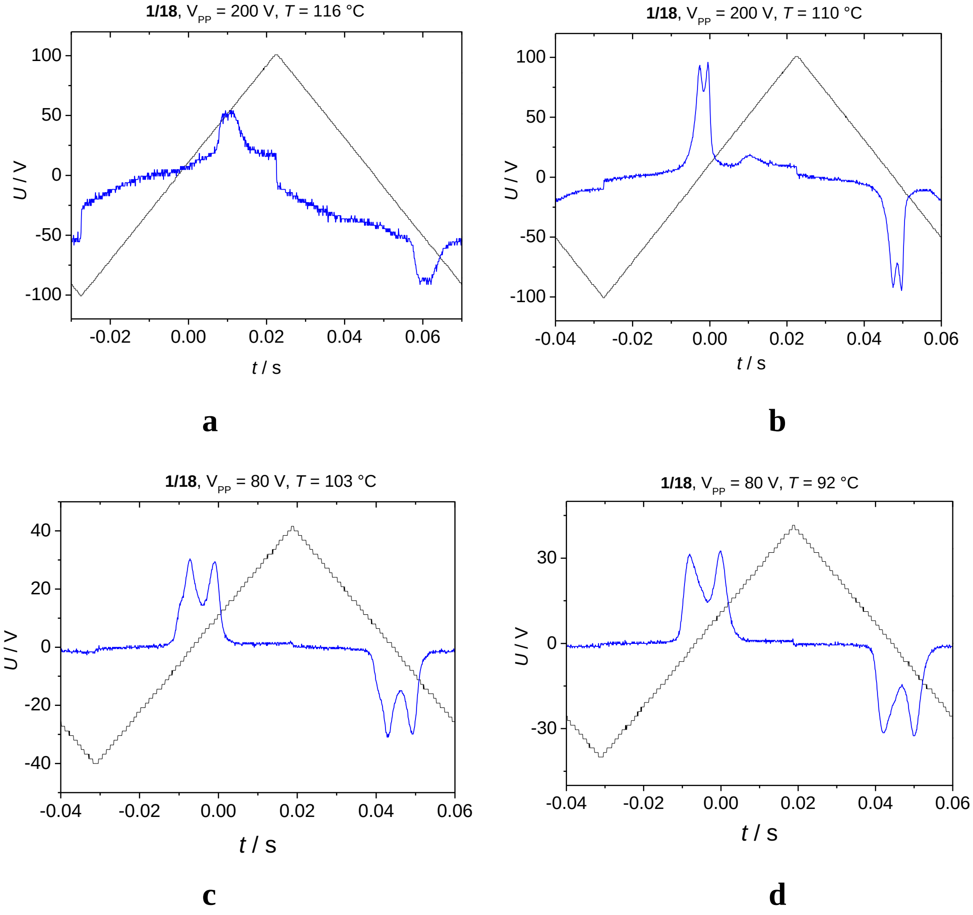

Supplementary Figure 6 │ Switching current response curves. Switching current obtained for **1/18** under a triangular wave voltage of 10 Hz: (**a**) in the SmC_s phase at 200 V_{pp} and (**b-d**) in the $SmC_sP_F/SmC_sP_F^{hel}$ phase region at 200/80 V_{pp}; for an explanation of the presence of two polarization current response peaks in the $SmC_sP_F^{hel}$ phase, see Supplementary Note 1.

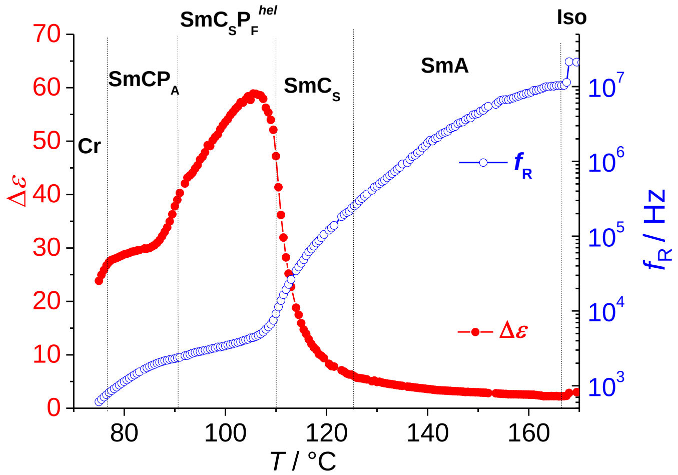

Supplementary Figure 7 │ Dielectric spectroscopic measurements. Dielectric relaxation strength ($\Delta\varepsilon$) and the relaxation frequency (f_R) as a function of temperature measured for **1/16** in a planar cell configuration, indicating the growth of polar domains at the SmC_S-$SmC_SP_F^{hel}$ transition as indicated by the exponential growths of $\Delta\varepsilon$.

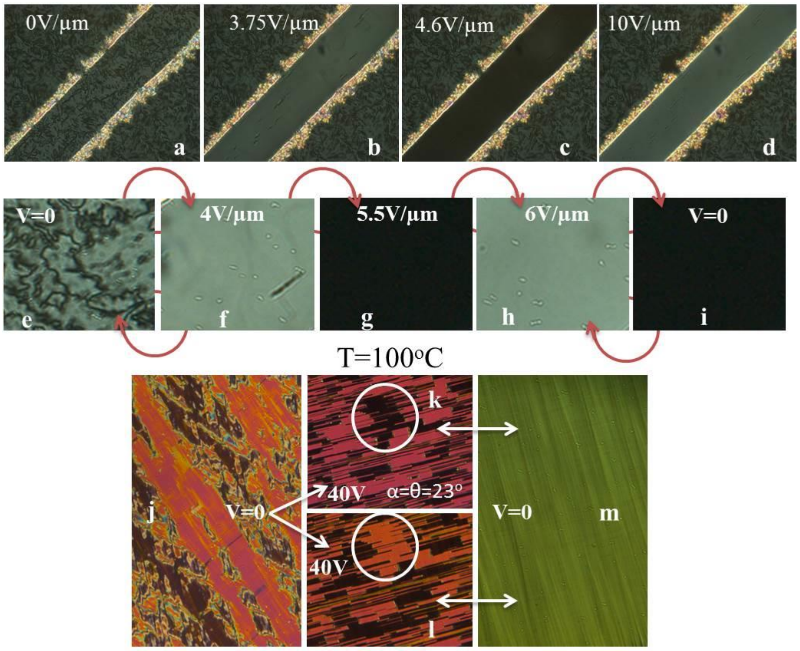

Supplementary Figure 8 │ Polarizing microscopic texture observation. Textures of **1/18** as observed on the application of an in-plane electric field of 110 Hz at 105 °C (**a-d**) on a 6.8 µm thick homeotropic cell. (**e-i**) shows field dependent optical switching in homeotropic (6.8 µm), and (**j-m**) planar (9 µm) cell configurations at 100 °C. (**k-l**) are the switching states for an alternating voltage signal.

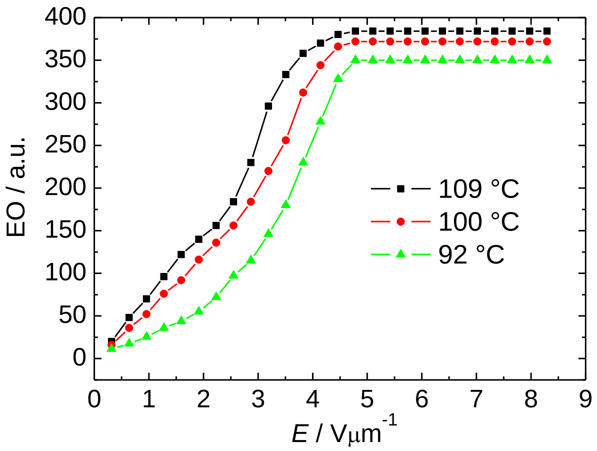

Supplementary Figure 9 │ Voltage dependence of electro-optical response. Measurement was performed in the $SmC_sP_F^{hel}$ phase of **1/18** filled in a planar cell (d = 9 µm) by keeping rubbing direction at an angle of 22.5° to the polarizer for three different temperatures: 109 °C, 100 °C and 92 °C.

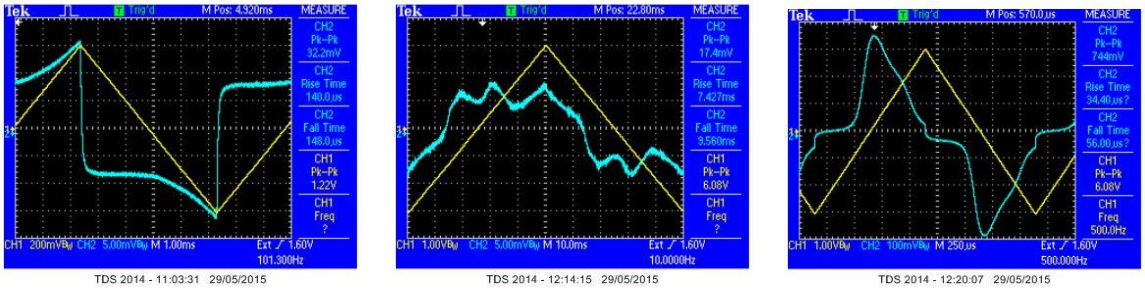

a **b** **c**

Supplementary Figure 10 │ Switching current response in a planar cell. The material **1/18** filled in a 6.3 µm planar cell and the switching currenr recorded at 110 °C to an applied triangular voltage of (**a**) 12 V, 100 Hz, (**b**) 60 V, 100 Hz, (**c**) 60 V, 500 Hz.

Suplementary Tables

Supplementary Table 1 │ Crystallographic data of compound 1/16 (θ is the Bragg angle).

	Small angle scattering		Wide angle scattering	
T/°C	θ/°	d/nm	θ/°	d/nm
160	0.849	5.200	9.195	0.482
155	0.837	5.277	9.230	0.481
150	0.827	5.343	9.250	0.480
145	0.818	5.401	9.280	0.478
140	0.810	5.452	9.305	0.477
135	0.805	5.485	9.320	0.476
130	0.800	5.524	9.350	0.475
125	0.792	5.578	9.380	0.473
120	0.784	5.637	9.395	0.472
115	0.774	5.705	9.435	0.470
110	0.764	5.780	9.460	0.469
105	0.755	5.853	9.500	0.467
100	0.745	5.929	9.535	0.465
95	0.735	6.013	9.565	0.464

Supplementary Table 2 │ Crystallographic data of compound 1/18 (θ is the Bragg angle).

	Small angle scattering		Wide angle scattering	
T/°C	θ/°	d/nm	θ/°	d/nm
155	0.847	5.217	9.369	0.474
150	0.834	5.299	9.379	0.473
145	0.823	5.367	9.415	0.471
140	0.814	5.425	9.434	0.470
135	0.806	5.481	9.457	0.469
130	0.801	5.517	9.477	0.468
125	0.795	5.557	9.518	0.466
120	0.785	5.624	9.554	0.464
115	0.768	5.752	9.557	0.464
110	0.762	5.799	9.576	0.463
105	0.752	5.871	9.607	0.462
100	0.741	5.959	9.637	0.460
95	0.734	6.020	9.675	0.459
90	0.724	6.101	9.706	0.457

Supplimentary Notes

Supplementary Note 1 │ Structure of the SmC_SP_F phase in relation to possible SmC_AP_A, SmC_α and de Vries structures. Polarization current response measurements in the phase designated as SmC_SP_F of **1/18** indicate the presence of two polarization current response peaks per half period of an applied triangular wave voltage (Supplementary Figures 6 and 10b). Electro-optical switching in $SmCP_A$ phases of bent-core molecules with transitions from initially a low-birefringent state with an apparent optical angle zero ($\theta_{app} = 0$) and the electric field induced higher birefringent state with non-zero apparent angle ($\theta_{app} > 0$) were observed and studied in a number of publications.[1-8] In these studies, the electro-optical response was assigned to the electric field induced transition from an anticlinic and low birefringent SmC_AP_A ground state to the higher birefringent synclinic SmC_SP_F state, leading to a tristable antiferroelectric switching in the ground state. This is usually supported by the observation of a double-peak switching current response to the applied triangular electric field; the technique commonly used methods to identifying ferroelectric/antiferroelectric phases.[9,10]

Double peak was also observed for switching in the SmC_SP_F phase reported here (Supplementary Figure 10b), but these peaks are relatively broad and are not clearly separated from each other, as has normally been observed in the antiferroelectric $SmCP_A$ phase[3]. Observation of the double switching peak may lead to erroneous conclusion due to the flow of an ionic current, [11] and/or the switching dynamics. For example, Niori *et al.* [12] first observed the ferroelectric-like single-peak switching current in bent-core system, which was later identified as antiferroelectric. The shape of switching current is also strongly dependent on the amplitude and frequency of the triangular voltage.[11] Supplementary Figure 10 presents the evolution of the shape of switching current response on parameters of the applied triangular voltage, amplitude and frequency for compound **1/18**.

On increasing the voltage and frequency, the shape of current response transforms to two peaks and then to one-peak. Also when the two-peaks are observed (Supplementary Figure 10b), these are not clearly separated from each other, as normally observed in the antiferroelectric phase.[12] Therefore, the switching current observation neither supports nor rejects the antiferroelectric nature of this phase. We therefore consider both the optical and electro-optical properties and these do not support the SmC_AP_A structure.

Supplementary References

1 Link, D. R., Natale, G., Shao, R., Maclennan, J. E., Clark, N. A., Korblova, E. & Walba, D. M. Spontaneous Formation of Macroscopic Chiral Domains in a Fluid Smectic Phase of Achiral Molecules. *Science* **278**, 1924-1927 (1997).

2 Heppke, G., Jakli, A., Rauch, S. & Sawade, H. Electric-field-induced chiral separation in liquid crystals. *Phys. Rev. E* **60**, 5575-5578 (1999).

3 Zennyoji, M., Takanishi, Y., Ishikawa, K., Thisayukta, J., Watanabe J. & Takezoe, H. Partial mixing of opposite chirality in a bent-shaped liquid crystal molecular system. *J. Mater. Chem.* **9**, 2775-2778 (1999).

4 Eremin, A., Nadasi, H., Pelzl, G., Diele, S., Kresse, H., Weissflog, W. & Grande, S. Paraelectric–antiferroelectric transitions in the bent-core liquid-crystalline materials. *Phys. Chem. Chem. Phys.* **6**, 1290-1298 (2004).

5 Shreenivasa Murthy, H. N., Bodyagin, M., Diele, S. Baumeister, U. Pelzl G. & Weissflog, W. Reentrant SmCP$_A$ phases: unusual polymorphism variant SmA–SmC$_S$P$_A$–Colo$_b$–SmC$_S$P$_A$ observed in new bent-core mesogens. *J. Mater. Chem.* **16**, 1634-1643 (2006).

6 Nakata, M., Chen, D., Shao, R., Korblova, E., Maclennan, J. E., Walba, D. M. & Clark, N.A. Electro-optic response of the anticlinic, antiferroelectric liquid crystal phase of a biaxial bent-core molecules with tilt angle near 45°. *Phys. Rev. E* 85, 031704 (2012).

7 Weissflog, W., Dunemann, U., Schröder, M. W., Diele, S., Pelzl, G., Kresse H. & Grande, S. Field-induced inversion of chirality in SmCP$_A$ phases of new achiral bent-core mesogens. *J. Mater. Chem.* **15**, 939-946 (2005).

8 Blinov, L. M., Barnik, M. I., Bustamante, E. S., Pelzl G. & Weissflog, W. Dynamics of electro-optical switching in the antiferroelectric B2 phase of an achiral bent-core shape compound. *Phys. Rev. E* **67**, 021706 (2004).

9 Fukuda, A., Takanishi, Y., Isozaki, T., Ishikawa K. & Takezoe, H. Antiferroelectric chiral smectic liquid crystals. *J. Mater. Chem.* **4**, 997-1016 (1994).

10 Takezoe, H., Gorecka, E. & Cepič, M. Antiferroelectric liquid crystals: Interplay of simplicity and complexity. *Rev. Mod. Phys.* **82**, 897-937 (2010).

11 Takezoe, H. & Takanishi, Y. Bent-Core Liquid Crystals: Their Mysterious and Attractive World. *Jpn. J. Appl. Phys*. **45**, 597-625 (2006).

12 Niori, T., Sekine, T., Watanabe, J., Furukawa T. & Takezoe, H. Distinct ferroelectric smectic liquid crystals consisting of banana shaped achiral molecules. *J. Mater. Chem.* **6**, 1231-1233 (1996).
